# Systemic sclerosis and risk of cardiovascular disease: A PRISMA-compliant systemic review and meta-analysis of cohort studies

**DOI:** 10.1097/MD.0000000000023009

**Published:** 2020-11-20

**Authors:** Xintao Cen, Sining Feng, Shanshan Wei, Lu Yan, Ledong Sun

**Affiliations:** a Department of Dermatology, Zhujiang Hospital, Southern Medical University; b Department of Dermatology, the Fifth Affiliated Hospital of Southern Medical University, Guangzhou, China.

**Keywords:** association, cardiovascular disease, meta-analysis, systemic sclerosis

## Abstract

**Background:**

Systemic sclerosis (SSc) is an autoimmune disorder leading to extensive fibrosis and microvascular injury. Macrovascular disease is well documented in other autoimmune rheumatic diseases such as systemic lupus erythematosus and rheumatoid arthritis. However, the link is unclear between SSc and macrovascular disease, particularly atherosclerotic cardiovascular disease (CVD). This meta-analysis aimed to investigate the association between SSc and CVD.

**Methods:**

A thorough literature search was conducted in the Cochrane, Embase, Medline, and PubMed to identify all cohort studies comparing the risk of CVD with and without SSc. The pooled hazard ratios (HRs) with 95% confidence intervals (CIs) of cardiovascular end points were calculated. The risk of bias of included studies was assessed by the Newcastle-Ottawa scale.

**Results:**

Seven cohort studies with a total of 14,813 study participants were included. In a comparison of SSc patients versus non-SSc controls, the pooled HR for cardiovascular disease was 2.36 (95% CI 1.97–2.81); for peripheral vascular disease was 5.27 (95%CI 4.27–6.51); for myocardial infarction was 2.36 (95% CI 1.71–3.25); and for stroke was 1.52 (95% CI 1.18–1.96).

**Conclusion:**

This meta-analysis revealed that SSc was associated with an increased risk of CVD. Clinicians who manage patients with SSc should be aware of the increased cardiovascular burden and undertake preventive measures.

## 1 Introduction

Systemic sclerosis (SSc), also termed scleroderma, is a multisystem autoimmune disease affecting approximately 20 per million people.^[1]^ Women are at a much higher risk of developing SSc than men ranging from 3:1 to 14:1 and the average age at diagnosis is in the fifth life decade.^[2]^ The clinical presentation of SSc is characterized by Raynaud's phenomenon, skin thickening, and fibrosis of internal organs. Although the underlying pathology is unknown, autoimmune inflammation, fibrosis, and vasculopathy may be involved in the pathogenesis of SSc.^[3,4]^

Involvement of the microvasculature is a prominent feature of SSc. Etiological factors include endothelial injuries, immune activation, proliferative obliterative vascular lesions, and progressive loss of capillaries. Increased risk of microvascular diseases in SSc, including Raynaud's phenomenon, pulmonary arterial hypertension, and renal crisis, has been well established.^[3,5]^ Macrovascular impairment was not originally identified as a feature of SSc with manifestations such as myocardial infarction, stroke, and peripheral vascular disease. However, SSc patients have been shown to have decreased peripheral vascular reactivity and endothelial dysfunction compared with healthy controls.^[6]^ Several studies have revealed an increased prevalence of subclinical atherosclerosis in SSc via measurements of carotid intima-media thickness (CIMT) and flow-mediated dilatation (FMD) on Doppler ultrasound, and coronary artery calcification on multidetector CT.^[7–9]^ Previous meta-analysis reported a higher incidence of coronary artery disease among patients with SSc.^[10]^ In addition, other autoimmune rheumatic diseases such as rheumatoid arthritis and systemic lupus erythematosus have been linked with an increased risk of developing cardiovascular disease (CVD), predominantly due to accelerated atherosclerosis.^[11–14]^ The association between SSc and CVD, however, remains unclear.

Therefore, in this study, we aimed to quantitatively synthesize current evidence on the association of SSc with CVD using a meta-analysis of cohort studies.

## 2 Methods

In accordance with the Preferred Reporting Items for Systematic Reviews and Meta-analysis (PRISMA), we performed this meta-analysis of cohort studies on the association between SSc and risk of CVD.^[15]^

### 2.1 Search strategy

The Cochrane Library for literature, Embase, Medline, and PubMed were systematically searched from inception to October 14, 2019, without language or geographic restrictions. We used different combinations of terms “systemic sclerosis” OR “systemic scleroderma” and “cardiovascular disease” OR “peripheral vascular disease” OR “myocardial infarction” OR “stroke” and “cohort study.” We also screened the reference lists of all articles identified (see Table 1, Supplemental Content, which described the detailed search strategies of electronic databases). The study protocol was approved by the Ethics Committee of the Zhujiang Hospital of Southern Medical University.

### 2.2 Study selection

Two independent authors (XC and LY) screened the titles and abstracts of the search results. The remaining full text of articles was assessed for eligibility. Any disagreements were resolved by consensus. Studies were eligible for inclusion if they were cohort studies; reported a quantitative association between SSc and risk of CVD versus non-SSc; enrolled participants without a history of CVD; and had at least 1-year follow-up. Studies were excluded if they were case-control studies or cross-sectional studies; were case series, reviews, abstracts or conference articles; did not report on CVD outcomes of interest.

#### 2.2.1 Data extraction and assessment of risk of bias

Data were extracted independently by 2 authors (LY and SW), using a pilot-tested form. For each study, the following information was extracted: first author name, year of publication, country, study design, study period, participant characteristics, outcomes, outcome assessment, number of cardiovascular disease cases, and mean follow-up years. The risk of bias in cohort studies was assessed by the Newcastle-Ottawa scale.^[16]^

### 2.3 Statistical analyses

We chose to perform a meta-analysis on adjusted results from included studies. We used hazard ratio estimates that were fully adjusted for confounding factors (see Table 2, Supplemental Content, which lists all the covariates).

This statistical analysis was conducted using Review Manager software, version 5.3 (The Cochrane Collaboration) and Stata software, version 12.1 (StataCorp). We calculated pooled hazard ratios (HRs) with 95% confidence intervals (95% CIs) of cardiovascular end points in patients with SSc when compared with non-SSc controls. Specifically, these included cardiovascular disease, peripheral vascular disease, myocardial infarction, and stroke. A generic inverse-variance (IV) method was used to pool the data with a random-effects meta-analysis model. Statistical heterogeneity was quantified with the I-squared statistics for each end point category. Substantial heterogeneity was defined as an I^2^ value greater than 50%.^[17]^ Publication bias was evaluated by visual inspection of funnel plots and by identifying asymmetry of funnel plots using the Egger test.

## 3 Results

**Figure 1:**
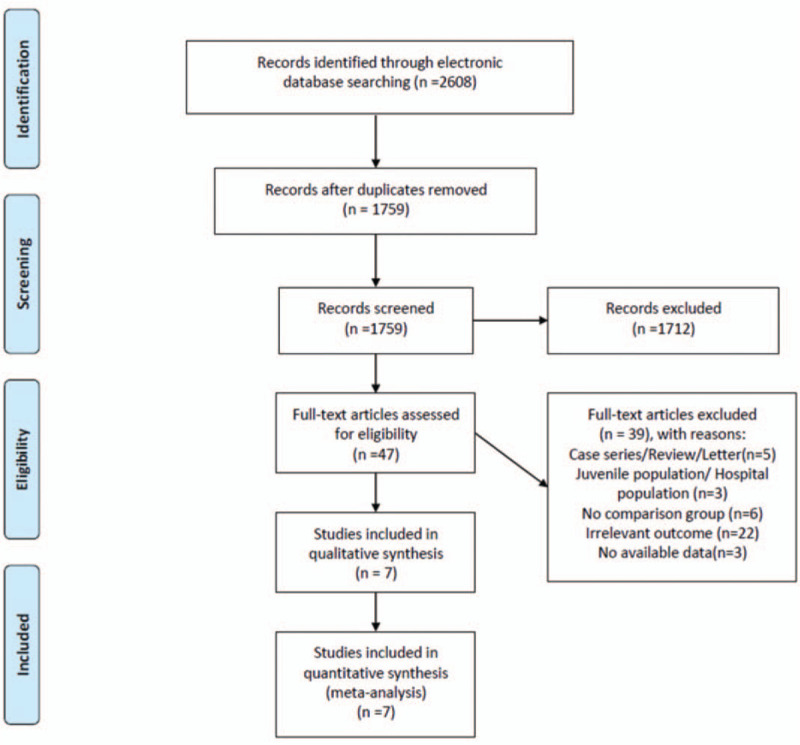
PRISMA study flow diagram.

The PRISMA study flow diagram is shown in Figure 1. The systematic literature search of articles published before October 14, 2019, identified 2608 articles, of which 2561 were deemed ineligible based on the titles and/or abstracts. After careful examination of the full text, 7 cohort studies that met the inclusion criteria were identified.

### 3.1 Study characteristics

The basic characteristics of the included cohort studies are presented in Table 1. Three studies were prospective and 4 were retrospective. Studies originated from Canada,^[18]^ USA,^[19,20]^ the UK,^[20]^ Denmark,^[21,22]^ and Taiwan.^[23,24]^ The 7 studies included a total of 14,813 SSc patients and 5,506,836 controls. The SSc cohorts had mean follow-up period that ranged from 4.3 to 5.2 years. The assessment of outcomes varied among these studies.

**Table 1:** Characteristics of cohort studies.

Reference	Study setting	Study design	Study period	SSc/Non-SSc	Mean age SSc/non-SSc	Female (%) SSc/non-SSc	Cases SSc/non-SSc	Outcomes	Outcome assessment	Mean follow-up years	NOS
Chiang,^[23]^ 2013	NHIRD, Taiwan	RC	1997–2006	1238/12,380	49.4/49.4	76/76	86/679	Stroke	ICD-9	4.7	8
Man,^[20]^ 2013	THIN, UK	RC	1986–2011	MI and stroke: 865/8643 PVD: 858/8580	58.7/58.7	85.8/85.8	MI:20/129 Stroke:22/129 PVD:34/96	MI, Stroke, PVD	OXMIS and Read codes	5.2	8
Chu,^[24]^ 2013	NHIRD, Taiwan	PC	1997–2006	1344/13,440	50.6/50.6	75.7/75.7	31/203	Acute MI	ICD-9	4.3	8
Avina-Zubieta,^[18]^ 2016	Population Data BC, Canada	PC	1996–2010	1223/12,433	56.1/56.1	83.2/83.1	MI:59/232 Stroke:37/212 CVD:84/406	MI, Stroke, CVD	ICD-9 or ICD-10	5	8
Hesselvig,^[21]^ 2018	Danish population	PC	1997–2011	1962/5,428,380	49.2/40.2	80/50.9	310/506,536	CVD	ICD-10	NR	7
Ying,^[19]^ 2019	VA Health System, USA	RC	1999–2014	4545/9090	60.9/61.0	17/17	353/574	Stroke	ICD-9	5.1	7
Butt,^[22]^ 2019	Danish administrative registries	RC	1995–2015	2778/13,890	55/55	76/76	MI:100/252 Stroke:112/439 PVD:184/182	MI, Stroke, PVD	ICD-8 or ICD-10	NR	8

BC = British Columbia, CVD = cardiovascular disease, ICD = International Classification of Diseases, MI = myocardial infarction, NHIRD = National Health Insurance Research Database, Non-SSc = no diagnostic codes for systemic sclerosis, NOS = Newcastle-Ottawa scale, NR = not reported, OXMIS = Oxford Medical Information System, PC = prospective cohort, PVD = peripheral vascular disease, RC = retrospective cohort, SSc = systemic sclerosis, THIN = The Health Improvement Network database, VA = Veterans Affairs.

### 3.2 Results of meta-analysis

#### 3.2.1 Association of SSc with cardiovascular disease

**Figure 2:**
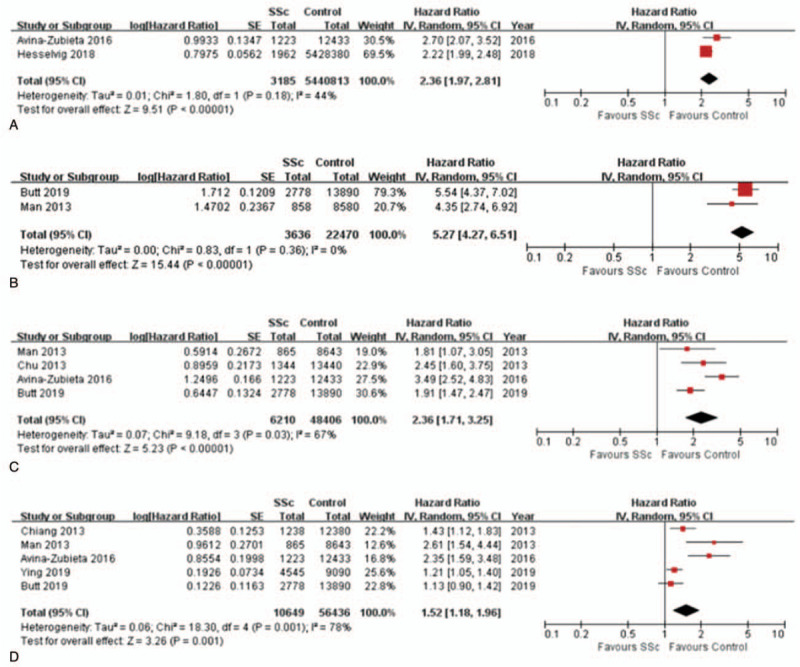
Forest plots of the association of systemic sclerosis (SSc) with cardiovascular diseases. A, The association between SSc and risk of cardiovascular disease. B, The association between SSc and risk of peripheral vascular disease. C, The association between SSc and risk of myocardial infarction. D, The association between SSc and risk of stroke. A generic inverse-variance (IV) method was used to calculate the pooled hazard ratio (HR) with a random-effects meta-analysis model. Heterogeneity was assessed using the I^2^ statistics. SE = standard error, CI = confidence interval.

Two cohort studies, including 3185 SSc patients, reported the association between SSc and CVD and these originated from Europe and North American. The meta-analysis showed that SSc was associated with a statistically significant increased risk of CVD (pooled HR 2.36, 95% CI 1.97–2.81), and mild heterogeneity was detected (I^2^ = 44%, *P* = .18) (see Fig. 2A).

#### 3.2.2 Association of SSc with peripheral vascular disease

Two cohort studies, including 3636 SSc patients, provided data on the association between SSc and peripheral vascular disease. These 2 studies originated from Europe. The results of the meta-analysis revealed that SSc significantly increased the risk of peripheral vascular disease (pooled HR 5.27, 95% CI 4.27–6.51). No statistically significant heterogeneity was found across these studies (I^2^ = 0%, *P* = .36) (see Fig. 2B).

#### 3.2.3 Association of SSc with myocardial infarction

Four cohort studies, including 6210 SSc patients, investigated the association between SSc and myocardial infarction. Two of these studies originated from Europe, 1 from North American, and 1 from Asia. The random-effects model meta-analysis showed that SSc was associated with a significantly increased risk of myocardial infarction (pooled HR 2.36, 95% CI 1.71–3.25). Substantial statistical heterogeneity was observed across these studies (I^2^ = 67%, *P* = .03) (see Fig. 2C).

#### 3.2.4 Association of SSc with stroke

Five cohort studies, including 10,649 SSc patients, evaluated the association between SSc and stroke. Two of these studies originated from Europe, 2 from North American, and 1 from Asia. The random-effects model meta-analysis illustrated an increased risk of stroke in patients with SSc (pooled HR 1.52, 95% CI 1.18–1.96). There was substantial statistical heterogeneity between studies (I^2^ = 78%, *P* = .001) (see Fig. 2D).

#### 3.2.5 Risk of bias and publication of bias assessment

**Figure 3:**
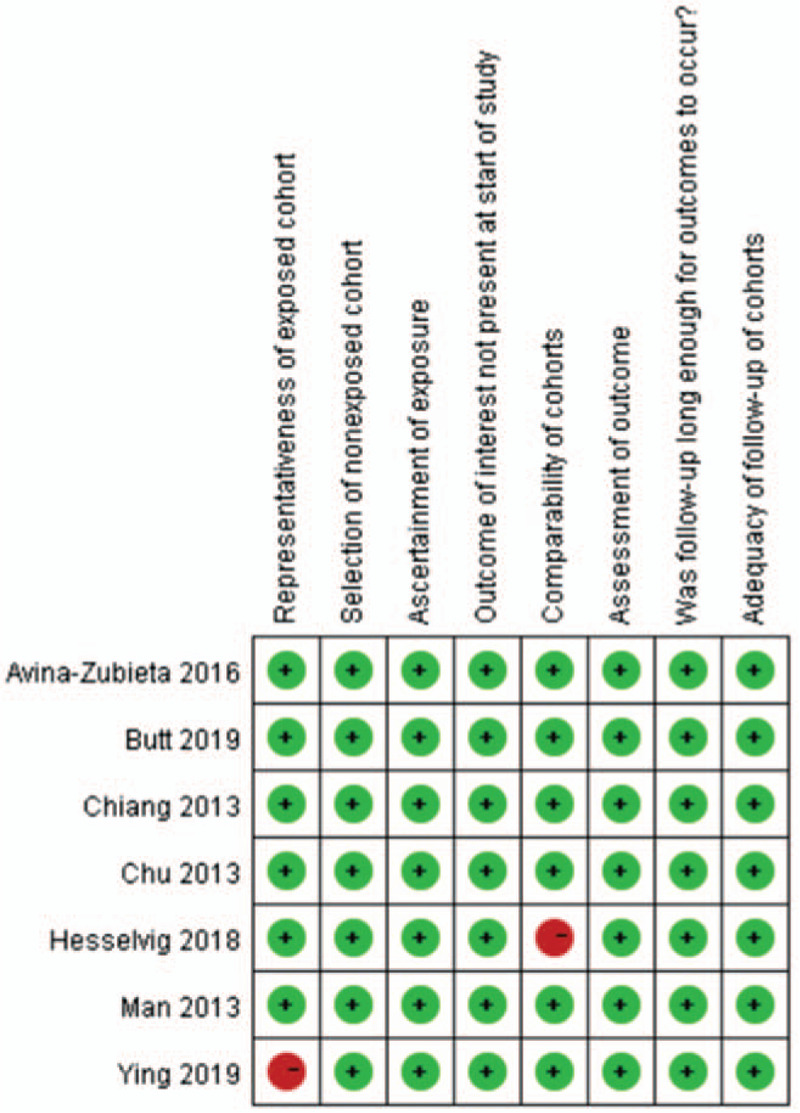
Risk of bias of the cohort studies. Risk of bias of cohort studies was assessed by the Newcastle-Ottawa scale. Green color represents low risk of bias, red for unclear risk of bias.

The risk of bias among included cohort studies was summarized in Figure 3. Of the 7 included studies, 5 were at low risk of bias and 2 were at high risk. As to the representativeness of exposed cohort, the study by Ying et al was rated with a high risk because the SSc samples were derived from a group of veterans.^[19]^ As to the comparability of cohorts, the study by Hesselvig et al was rated with a high risk of bias because the age and sex were not matched between SSc patients and control cohorts.^[21]^ All 7 included cohort studies were determined to have a low risk of bias in the domain of adequacy of follow-up of cohorts because the durations of follow-up exceeded 1 year.

The funnel plots evaluated the association of CVD and patients with SSc. In our study, the shape of funnel plots appeared to be symmetrical. The results of Egger test showed no evidence for publication bias (see Figure 1, Supplemental Content, which detects publication bias).

## 4 Discussion

In this meta-analysis of 14,813 study participants from 7 cohorts, SSc was significantly associated with higher risks of cardiovascular disease, peripheral vascular disease, myocardial infarction, and stroke.

Our study indicated that patients with SSc were prone to have comorbid CVD. A previously published meta-analysis investigated the risk of stroke in patients with SSc.^[25]^ In this work, Ungprasert et al analyzed 4 cohort studies and found a significant association between SSc and increased risk of stroke (RR 1.68, 95% CI 1.26–2.24). However, some limitation to select data in their study should be noted. They included the study by Zoller et al^[26]^ using hospital-based data, which may lead to potential selection bias. Considering several limitations of Ungprasert's study, our meta-analysis provides more precise estimates using nation- or state-wide data.

Most of the included cohort studies were deemed to have a low risk of bias according to the Newcastle-Ottawa Scale. We evaluated the two included studies as having a high risk of bias because of study participants from a specific group and no control for confounder factors.^[19,21]^ Consequently, to confirm the association between SSc and CVD more exactly, these points in study design should be taken into account in future prospective cohort studies.

The mechanisms for the increased risk of atherosclerotic cardiovascular disease in patients with SSc are far from being fully elucidated. One possible mechanism is inflammation.^[27]^ SSc has been characterized by T-cell activation and autoantibody formation. Various pro-inflammatory cytokines that have been associated with both atherosclerotic plaques and CIMT, including interleukin (IL)-2, IL-6, and C-reactive protein, have been shown to be increased in patients with SSc.^[9,28]^ Furthermore, emerging studies have found that autoantibodies may have a role in atherogenesis, including those against oxidized low-density lipoproteins (ox-LDL), β2-glycoprotein-I (β2GPI), centromere and cardiolipin.^[29,30]^ Anti-centromere antibody (ACA) is one of the specific hallmark antinuclear antibodies (ANAs) of SSc. Ischemic macrovascular events and plaques are more common in the ACA positive SSc patients when compared with both ACA negative SSc patients and controls.^[31]^

Another possible mechanism is endothelial dysfunction, which is the key event in the pathophysiology of SSc and atherosclerosis. Although the original stimulus is unknown, endothelial cell injury is regarded as a main driver of vascular alterations, increased matrix deposition and excess fibrous tissues characterizing SSc. Activated endothelial cells express adhesion molecules that promote the recruitment and transmigration of inflammatory cells into vessel walls and infiltration of the extracellular matrix.^[32]^ In addition, several evidences have suggested that high levels of reactive oxygen species (ROS) and oxidative stress caused by endothelial dysfunction and vascular inflammation, was observed in SSc, leading to oxidation of LDL. Accumulation of oxidized low-density lipoproteins triggers endothelial cell activation and further amplifies inflammatory processes.^[8,33,34]^ The derangement of vasoactive mediators has also been suggested to be an essential component of endothelial dysfunction in SSc, with upregulation of vasoconstrictive endothelin and downregulation of the vasodilator nitric oxide.^[5]^ Inflammatory vascular injury, intimal proliferation and fibrosis may result in the formation and development of atherosclerotic plaques.^[35,36]^ Thus, inflammation and endothelial dysfunction may contribute to the pathogenesis of vascular abnormalities in atherosclerosis and SSc.

This study has several limitations. Firstly, there was substantial statistical heterogeneity with regard to the outcomes of stroke and myocardial infarction, given the differences in baseline characteristics such as gender, age and SSc history. Although we used random-effects models and pooled only studies with similar outcomes, it did not eliminate heterogeneity. Secondly, none of the primary studies provided information on disease severity of SSc. However, because of the evolving classification criteria and the growing use of serum markers and capillaroscopy, patients fulfilling SSc criteria have increased.^[37–39]^ Compared with earlier cohorts, later cohorts were more likely to contain a larger number of patients with early mild SSc, which may tend to bias the results in the assessment of CVD risk.^[22]^ Thirdly, there was some evidence that the rate of cardiac involvement in diffuse cutaneous SSc was higher than that in limited cutaneous SSc.^[40]^ However, we could not evaluate the risks for the two major disease subtypes because no studies examined the association between different subtypes of SSc and CVD. Fourthly, most studies were conducted in Caucasian populations. Several evidences have revealed that SSc in blacks had more severe clinical manifestations and greater mortality than in whites.^[41,42]^ Considering racial differences, more studies in other ethnicities such as Afro-Americans and Asians are needed. Finally, we could not rule out the possibility of residual confounding because no data was available on traditional cardiovascular risk factors, such as body mass index, smoking and family history of CVD.

Despite these limitations, this study has some strengths. First, to our knowledge, this is the largest study of adults with SSc and long-term follow-up of cardiovascular outcomes. Second, only large-scale cohort studies providing more definitive evidence were included. We excluded cross-sectional and case-control studies, which highly reduced the likelihood of recall bias and selection bias. Third, considering the degree of risk, a meta-analysis provides greater statistical power to detect potential associations in comparison with individual studies alone.

In conclusion, this meta-analysis confirms a significant association of SSc with CVD. Future studies should focus on identifying risk factors for CVD in SSc, such as clinical subtypes or disease severity. Furthermore, clinicians who manage patients with SSc should be aware of the increased risk of CVD from the time of SSc diagnosis and undertake preventive measures.

## Author contributions

LS conceived and designed the study. XC and LY did the literature review. LY and SW extracted the data and performed the analysis. XC and LY wrote and edited the manuscript. All authors revised the manuscript and provided approval of the final version.

**Conceptualization:** Ledong Sun.

**Data curation:** Shanshan Wei, Lu Yan.

**Formal analysis:** Shanshan Wei, Lu Yan.

**Investigation:** Xintao Cen, Lu Yan.

**Software:** Shanshan Wei, Lu Yan.

**Supervision:** Ledong Sun.

**Writing – original draft:** Xintao Cen, Sining Feng.

**Writing – review & editing:** Xintao Cen, Sining Feng.

## Supplementary Material

Supplemental Digital Content
