# Supplementary material for: Systemic sclerosis and risk of cardiovascular disease: A PRISMA-compliant systemic review and meta-analysis of cohort studies
Source: Medicine (Baltimore). 2020 Nov 20;99(47):e23009. doi: 10.1097/MD.0000000000023009 (PMC7676589; doi:10.1097/MD.0000000000023009)
Supplement: Supplemental Digital Content [file medi-99-e23009-s001.pdf]

Electronic supplement material

Supplementary table 1 Search strategies of electronic database

**a. Search strategy used in CENTRAL**	
#1	MeSH descriptor: [Scleroderma, Systemic] explode all trees
#2	(systemic sclerosis*):ti,ab,kw
#3	(systemic scleroderma*):ti,ab,kw
#4	(systemic*):ti,ab,kw AND (sclerosis*):ti,ab,kw
#5	(sclerosis*):ti,ab,kw AND (systemic*):ti,ab,kw
#6	(systemic*):ti,ab,kw AND (scleroderma*):ti,ab,kw
#7	(scleroderma*):ti,ab,kw AND (systemic*):ti,ab,kw
#8	#1 or #2 or #3 or #4 or #5 or #6 or #7
#9	MeSH descriptor: [Cardiovascular Diseases] explode all trees
#10	(cardiovascular disease):ti,ab,kw
#11	(cardiovascular abnormalities):ti,ab,kw
#12	MeSH descriptor: [Myocardial Infarction] explode all trees
#13	(Myocardial infarction):ti,ab,kw
#14	(Myocardial Ischemia):ti,ab,kw
#15	MeSH descriptor: [Stroke] explode all trees
#16	(stroke):ti,ab,kw
#17	(cerebrovascular accident):ti,ab,kw
#18	(cerebrovascular disorder):ti,ab,kw
#19	MeSH descriptor: [Peripheral Vascular Diseases] explode all trees
#20	(peripheral vascular disease):ti,ab,kw
#21	#9 or #10 or #11 or #12 or #13 or #14 or #15 or #16 or #17 or #18 or #19 or #20
#22	MeSH descriptor: [Cohort Studies] explode all trees
#23	(cohort studies):ti,ab,kw
#24	#22 or #23
#25	#8 and #21 and #24

b. Search strategy used in EMBASE	
#1	systemic sclerosis':ab,ti
#2	systemic scleroderma':ab,ti
#3	systemic:ab,ti AND sclerosis:ab,ti
#4	sclerosis:ab,ti AND systemic:ab,ti
#5	systemic:ab,ti AND scleroderma:ab,ti
#6	scleroderma:ab,ti AND systemic:ab,ti
#7	systemic sclerosis'/exp OR 'systemic sclerosis' OR (systemic AND ('sclerosis'/exp OR sclerosis))
#8	#1 OR #2 OR #3 OR #4 OR #5 OR #6 OR #7
#9	cardiovascular disease'/exp OR 'cardiovascular disease' OR (('cardiovascular'/exp OR cardiovascular) AND ('disease'/exp OR disease))
#10	cardiovascular disease':ab,ti
#11	'cardiovascular abnormalities':ab,ti
#12	myocardial infarction'/exp OR 'myocardial infarction' OR (myocardial AND ('infarction'/exp OR infarction))
#13	myocardial infarction':ab,ti
#14	myocardial ischemia':ab,ti
#15	'stroke'/exp OR stroke
#16	stroke:ab,ti
#17	cerebrovascular accident':ab,ti
#18	cerebrovascular disorder':ab,ti
#19	peripheral vascular diseases'/exp OR 'peripheral vascular diseases' OR (peripheral AND vascular AND ('diseases'/exp OR diseases))
#20	'peripheral vascular diseases':ab,ti
#21	#9 OR #10 OR #11 OR #12 OR #13 OR #14 OR #15 OR #16 OR #17 OR #18 OR #19 OR #20
#22	cohort study'/exp OR 'cohort study' OR (cohort AND ('study'/exp OR study))
#23	'cohort study':ab,ti
#24	#22 OR #23
#25	#8 AND #21 AND #24

**c.**	**Search strategy used in MEDLINE**
#1	exp *Scleroderma, Systemic/
#2	systemic sclerosis.mp.
#3	systemic scleroderma.mp.
#4	systemic$ sclerosis.mp.
#5	sclerosis$ systemic.mp.
#6	systemic$ scleroderma.mp.
#7	scleroderma$ systemic.mp.
#8	1 or 2 or 3 or 4 or 5 or 6 or 7
#9	exp *Cardiovascular Diseases/
#10	cardiovascular disease.mp.
#11	cardiovascular abnormalities.mp.
#12	exp *Myocardial Infarction/
#13	myocardial infarction.mp.
#14	myocardial ischemia.mp.
#15	exp *Stroke/
#16	stroke.mp.
#17	cerebrovascular accident.mp.
#18	cerebrovascular disorder.mp.
#19	exp *Peripheral Vascular Diseases/
#20	peripheral vascular disease.mp.
#21	9 or 10 or 11 or 12 or 13 or 14 or 15 or 16 or 17 or 18 or 19 or 20
#22	exp *Cohort Studies/
#23	cohort study.mp.
#24	22 or 23
#25	8 and 21 and 24

Supplementary table 2 Detail of adjustment for studies in the meta-analysis

a. HRs of stroke

Study	Unadjusted HR (95%CI)	No adjustment	Fully adjusted HR (95%CI)	Adjustment for covariates
Avina-Zubieta ,2016	3.08 (2.14-4.44)	Matched on age, sex, entry time	2.35 (1.59-3.48)	Age, sex, entry time, number of outpatient visits, health resource utilization, glucocorticoids, comorbidities
Chiang, 2013	1.44 (1.15-1.80)	Matched on age, sex, entry time, comorbidity	1.43 (1.12-1.83)	Age, sex, entry time, comorbidities (hypertension, diabetes, dyslipidemia, chronic kidney disease, coronary artery disease, atrial fibrillation)
Man, 2013	2.56 (1.58-4.14)	Matched on age, sex, entry time	2.61 (1.54-4.44)	Age, sex, entry time, BMI, smoking, medication (aspirin, NSAID and oral glucocorticoid), comorbidities (hypertension, diabetes, hyperlipidemia, atrial fibrillation)
Ying, 2019	1.28 (1.11-1.47)	Matched on age, sex, race, smoking, status, VA site	1.21 (1.05-1.40)	Age, sex, race, smoking, status, VA site, medication (aspirin, non-steroidal anti-inflammatory drugs, statins), comorbidities (atrial fibrillation, hypertension, diabetes mellitus, non-cerebrovascular atherosclerotic disease, hyperlipidemia), Medicare enrollment
Butt, 2019	1.28 (1.04-1.58)	Matched on age, sex	1.13 (0.90-1.42)	Age, sex, medication (ASA, NSAID, glucocorticoids, statins, OAC and platelet inhibitors), comorbidities

b. HRs of myocardial infarction

Study	Unadjusted HR (95%CI)	No adjustment	Fully adjusted HR (95%CI)	Adjustment for covariates
Avina-Zubieta , 2016	4.26 (3.17-5.73)	Matched on age, sex, entry time	3.49 (2.52-4.83)	Age, sex, entry time, number of outpatient visits, health resource utilization, glucocorticoids, diabetes medication, comorbidities
Chu, 2013	2.40 (1.57-3.67)	Matched on age, sex, comorbidity	2.45 (1.60-3.75)	Age, sex, comorbidities (hypertension, diabetes, coronary artery disease, dyslipidemia, atrial fibrillation, peripheral arterial occlusive disease, chronic obstructive pulmonary disease, and chronic kidney disease)

Man, 2013	1.97 (1.21-3.22)	Matched on age, sex, entry time	1.80 (1.07-3.05)	Age, sex, entry time, BMI, smoking, medication (aspirin, NSAID and oral glucocorticoid), comorbidities (hypertension, diabetes, hyperlipidemia)
Butt, 2019	2.08 (1.65-2.64)	Matched on age, sex	1.91 (1.47-2.47)	Age, sex, medication (ASA, NSAID, Glucocorticoids, statins, OAC and platelet inhibitors), comorbidities

c. HRs of cardiovascular disease

Study	Unadjusted HR (95%CI)	No adjustment	Fully adjusted HR (95%CI)	Adjustment for covariates
Avina-Zubieta, 2016	3.54 (2.77-4.53)	Matched on age, sex, entry time	2.70 (2.07-3.51)	Age, sex, entry time, number of outpatient visits, health resource utilization, glucocorticoids, comorbidities
Hesselvig, 2018	NA	NA	2.22 (1.99-2.48)	Age, calendar year, sex, socioeconomic status, medication, comorbidities

d. HRs of peripheral vascular disease

Study	Unadjusted HR (95%CI)	No adjustment	Fully adjusted HR (95%CI)	Adjustment for covariates
Man, 2013	4.57 (2.99-7.01)	Matched on age, sex, entry time	4.35 (2.74-6.93)	Age, sex, entry time, BMI, smoking, medication (aspirin, NSAID and oral glucocorticoid), comorbidities (hypertension, diabetes, hyperlipidemia, atrial fibrillation)
Butt, 2019	5.73 (4.63-7.09)	Matched on age, sex	5.54 (4.37-7.02)	Age, sex, medication (ASA, NSAID, Glucocorticoids, statins, OAC and platelet inhibitors), comorbidities

Supplementary figure 1. Publication bias assessment

a. Funnel plot of the association between SSc and stroke

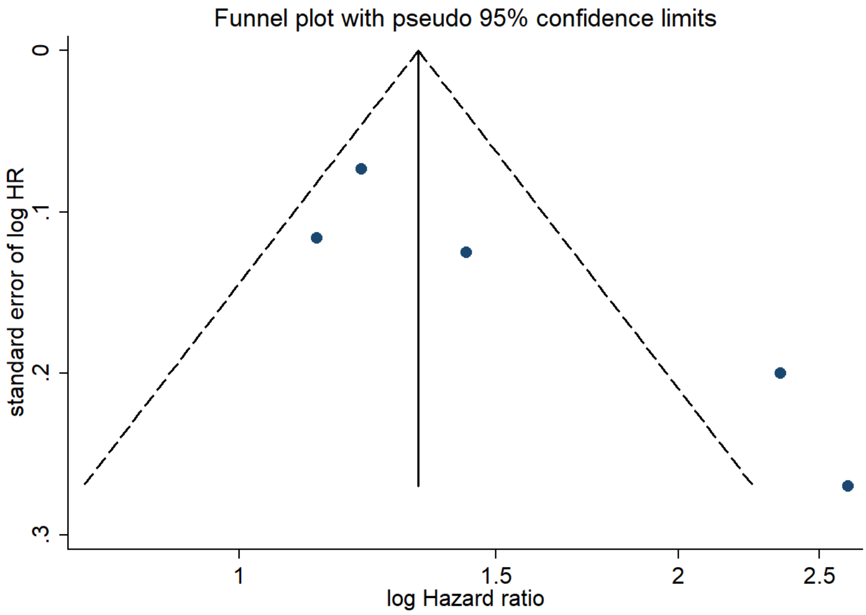

Egger's test: bias,4.15; 95%CI, -0.44 to 8.33; P=0.051.

b. Funnel plot of the association between SSc and myocardial infarction

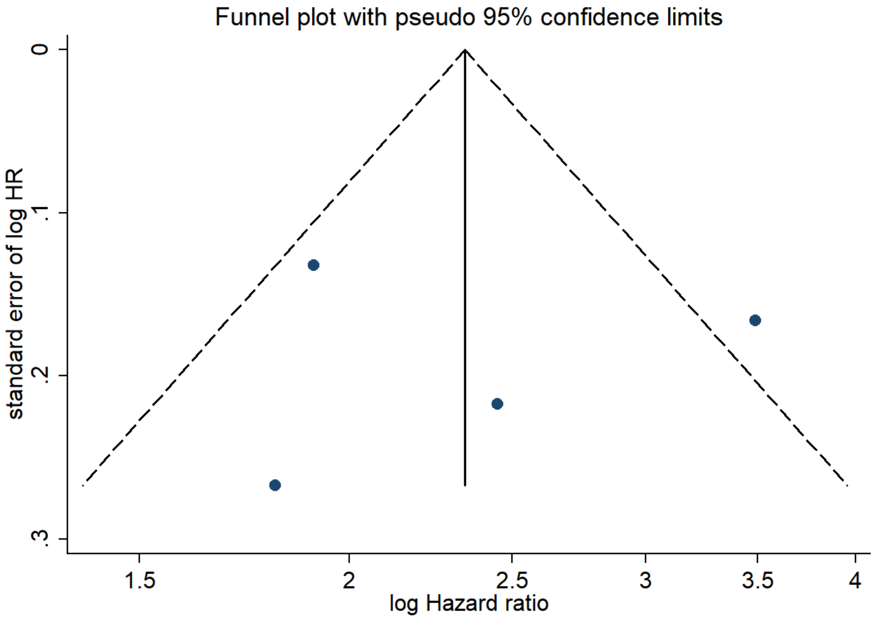

Egger's test: bias,0.29; 95%CI, -17.73 to 18.31; P=0.951.
